# Photoautotrophic Production of 2-O-α-D-Glucosylglycerol by Marine *Cyanobacterium aponinum* SCSIO-45682: Multi-Factor Optimization and Functional Evaluation

**DOI:** 10.3390/md24090327

**Published:** 2026-09-17

**Authors:** Yaqi Geng, Jingxue Ma, Weinan Wang, Lingyu Ouyang, Bingqi Xu, Hualian Wu, Houbo Wu, Pinghuai Liu, Tao Li, Wenzhou Xiang

**Affiliations:** 1 State Key Laboratory of Breeding Biotechnology and Sustainable Aquaculture, Guangdong Key Laboratory of Marine Materia Medica, South China Sea Institute of Oceanology, Chinese Academy of Sciences, Guangzhou 510301, China; gengyaqi24@mails.ucas.ac.cn (Y.G.); wangweinan0220@163.com (W.W.); ouyanglingyu24@mails.ucas.ac.cn (L.O.); xubingqi24@mails.ucas.ac.cn (B.X.); hlwu@scsio.ac.cn (H.W.); wuhoubo@scsio.ac.cn (H.W.); 2 University of Chinese Academy of Sciences, Beijing 101408, China; 3 School of Life and Health Sciences, Hainan University, Haikou 570228, China; majingxue@hainanu.edu.cn (J.M.); 4 School of Chemistry and Chemical Engineering, Hainan University, Haikou 570228, China; twlph@163.com (P.L.)

**Keywords:** *Cyanobacterium aponinum*, glucosylglycerol, culture optimization, response surface methodology, photoautotrophic cultivation

## Abstract

Glucosylglycerol (GG) is a compatible solute with excellent moisturizing capacity and macromolecule stability, exhibiting broad application potential in cosmetics, food, and pharmaceutical industries. The halophilic cyanobacterium *Cyanobacterium aponinum* SCSIO-45682 can synthesize GG under salt stress and represents a promising strain for photoautotrophic GG production. In this study, the GG product extracted from SCSIO-45682 was structurally identified as 2-O-α-D-GG by high-performance liquid chromatography (HPLC) and nuclear magnetic resonance (NMR) analysis. The effects of salinity, initial pH, light intensity, and carbon, nitrogen, and phosphorus concentrations on biomass and GG accumulation were systematically investigated using single-factor experiments, and response surface methodology (RSM) was subsequently applied to optimize intracellular GG content as a percentage of dry weight (% DW). The results showed that salinity was the primary factor driving GG accumulation in SCSIO-45682. As salinity increased from 30 ppt to 120 ppt, GG content increased by 3.41-fold. Light intensity was another key factor affecting GG accumulation, with moderate irradiance of 2000–5000 lux being more favorable for GG accumulation. Among the nutritional factors, nitrogen and phosphorus had relatively weak effects, whereas increasing carbon concentration enhanced GG yield by promoting biomass accumulation. The RSM results showed that salinity × light intensity exhibited a synergistic enhancement pattern, whereas the pH × carbon concentration interaction displayed an inverse regulatory effect. Under the optimal conditions of 96 ppt salinity, pH 5.0, 5000 lux light intensity, and 7.0 mM NaHCO_3_, GG content and yield reached 16.30% DW and 366.75 mg/L, respectively, representing a 3.29-fold increase compared with the control. In addition, the GG-containing crude extract exhibited concentration-dependent 2,2-diphenyl-1-picrylhydrazyl (DPPH) and 2,2′-azino-bis(3-ethylbenzothiazoline-6-sulfonic acid, ABTS) radical-scavenging activities. Overall, this study achieved a high level of natural 2-O-α-D-GG accumulation in a wild-type cyanobacterial photoautotrophic cultivation system through multi-factor synergistic optimization, providing a foundation for the green biomanufacturing of GG using wild-type *C*. *aponinum*.

## 1. Introduction

Marine cyanobacteria are increasingly recognized as valuable sources of metabolites with biotechnological, cosmeceutical, nutritional, and pharmaceutical potential [1,2]. The accumulation of compatible solutes represents a key strategy for cyanobacterial adaptation to high-salinity marine environments. As water-soluble and low-molecular-weight compounds, they can accumulate to high intracellular concentrations without disrupting cellular metabolism, thereby enabling cyanobacteria to survive osmotic stresses such as hypersalinity and desiccation [3,4]. The common compatible solutes from cyanobacteria include glycerol, glucosylglycerol (GG), glycine betaine, and trehalose [3,5]. Beyond their physiological role for cyanobacterial cells in osmotic acclimation, some compatible solutes also exhibit considerable commercial potential due to their excellent biocompatibility and stability. One notable example is GG, which has attracted increasing attention in cosmetics, food, and pharmaceutical industries [6,7].

GG is a small-molecule heteroside consisting of one glycerol molecule and one glucose moiety linked by a glycosidic bond [8]. According to the anomeric configuration of the glycosidic linkage, naturally occurring GG can be mainly classified into β-GG and α-GG [3]. β-GG is found mainly in higher plants, while α-GG, particularly 2-O-α-D-GG, is widespread in cyanobacteria as a compatible solute [5]. Among the GG isomers identified to date, 2-O-α-D-GG has received the most attention because its functional properties and commercial value have been well demonstrated. Owing to its exceptional water-holding capacity, anti-aging effects, and robust macromolecule-stabilizing properties, GG demonstrates great commercial value as a premium moisturizing agent in cosmeceuticals [8,9], a low-calorie sweetener or protein stabilizer in food technology, and a protective matrix for enzyme preservation in pharmaceuticals [10]. Studies also indicate that GG improves skin hydration, barrier function, and elasticity, with antioxidant activity relevant to anti-aging [11,12].

Despite its broad application prospects, efficient production of natural GG remains a major challenge. In cyanobacteria, salinity is well established as a major environmental trigger for GG biosynthesis [13,14], and the molecular basis of salt-induced GG accumulation has been characterized in detail in *Synechocystis* sp. PCC 6803 [3,5,6,15]. Similar salinity-dependent responses have also been reported in *Synechococcus* sp. PCC 7002 [16] and *Arthrospira maxima* [17], indicating that salt-induced GG accumulation represents a widespread osmotic acclimation strategy among GG-producing cyanobacteria. Building on this physiological basis, various biotechnological strategies have been developed to improve GG production. Engineered *Synechocystis* sp. PCC 6803 combined with gel encapsulation achieved a cumulative GG yield of 1640 mg/L under semi-continuous operation [18], while heterologous expression of the GgpS/GgpP pathway in *Corynebacterium glutamicum* yielded approximately 2000 mg/L [19]. Enzymatic and whole-cell catalytic systems based on sucrose phosphorylase have achieved substantially higher volumetric yield of 65–452 g/L (Table 1), but these approaches require externally supplied organic substrates such as sucrose and glycerol rather than relying on photoautotrophic carbon fixation [20,21,22,23]. These production strategies generally entail higher production costs and greater process complexity, which may limit their economic feasibility for large-scale sustainable GG production.

Wild-type photoautotrophic cyanobacteria provide an alternative route for GG production using light and inorganic carbon as the principal inputs, but this strategy remains comparatively less developed. Wild-type *Synechocystis* sp. PCC 6803 produces approximately 100 mg/L GG under salt-stressed batch conditions [18], whereas photoautotrophic cultivation of wild-type *A*. *maxima* achieved a GG content of 15.77% DW and a yield of 189.99 mg/L [17]. Previous studies on cyanobacterial GG accumulation have predominantly emphasized salinity responses and their underlying molecular regulation, whereas the combined effects and interactions of multiple cultivation factors remain less systematically investigated. Among potential wild-type photoautotrophic production strains, *Cyanobacterium aponinum* is of particular interest because of its broad environmental tolerance. This cyanobacterium can grow at high temperatures (30–45 °C), under alkaline conditions, and at high salinity [24,25], and several *C*. *aponinum* strains have shown potential for biomass production and the accumulation of high-value compounds [26,27,28]. The marine-derived strain *C*. *aponinum* SCSIO-45682 therefore represents a promising platform for photoautotrophic GG production. However, the effects and interactions of multiple cultivation factors on GG accumulation in this strain, the structural identity of the naturally accumulated GG product, and the radical-scavenging properties of GG-containing extracts remain insufficiently characterized. Addressing these questions would extend previous salinity-focused studies toward an integrated evaluation of wild-type photoautotrophic GG production.

This study aimed to establish a multi-factor optimization framework for intracellular 2-O-α-D-GG accumulation under photoautotrophic cultivation using wild-type *C. aponinum* SCSIO-45682 and to evaluate the functional properties of the extracted product. To this end, the GG accumulated by SCSIO-45682 was structurally characterized by HPLC and NMR to confirm its identity as 2-O-α-D-GG. The effects of salinity, initial pH, light intensity, and nutrient supply on biomass and GG accumulation were then systematically investigated through single-factor experiments, and response surface methodology was subsequently applied to characterize multi-factor interactions and identify the cultivation conditions that maximized intracellular GG content (% DW). The biochemical composition of the biomass under the optimized conditions and the radical-scavenging activity of the GG-containing crude extract were also assessed.

## 2. Results

### 2.1. Structural Identification of the Extracted Product as 2-O-α-D-Glucosylglycerol

**Figure 1:**
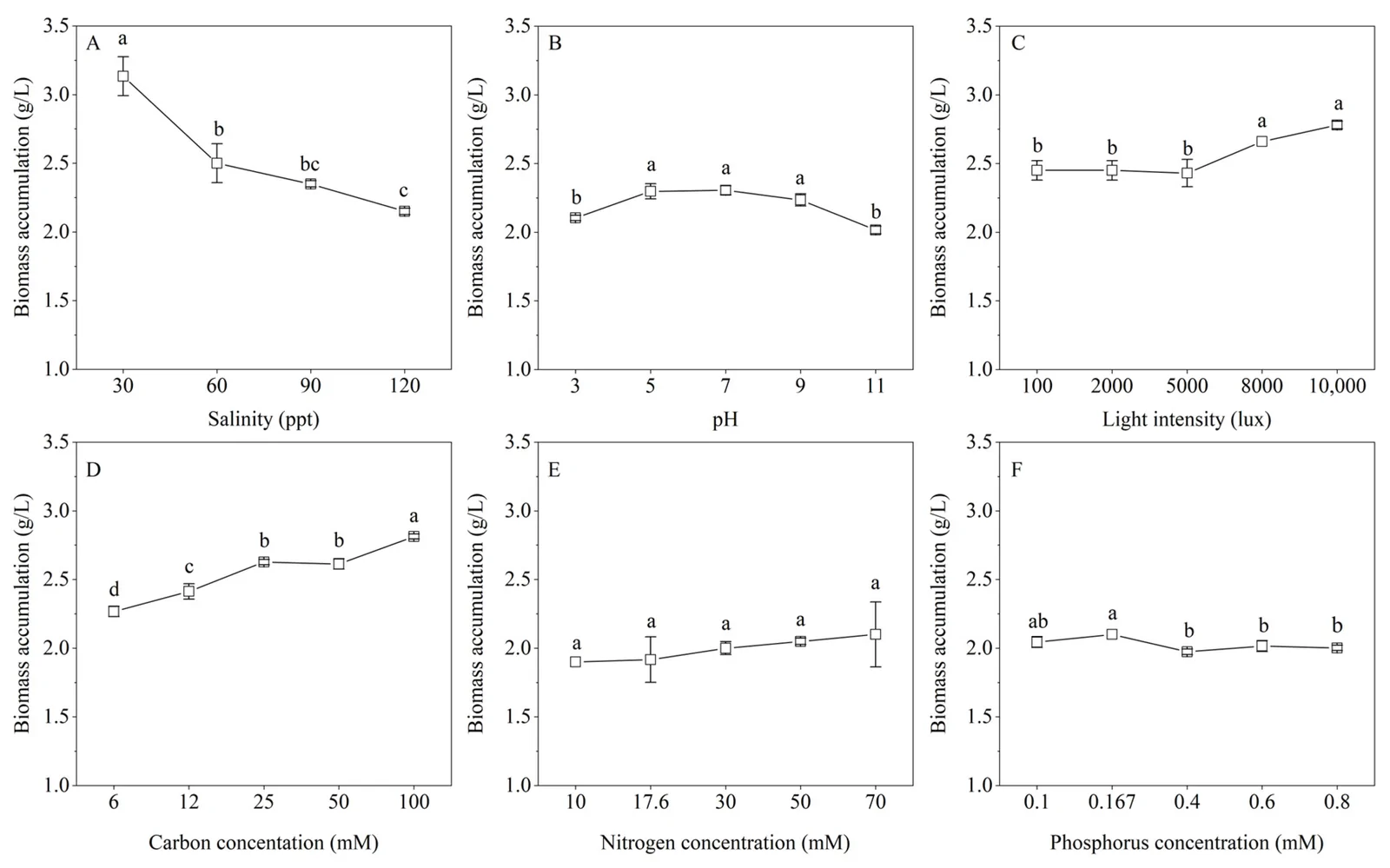
HPLC chromatograms and NMR spectra of the GG sample and the 2-O-α-D-glucosylglycerol (2-O-α-D-GG) standard. (**A**) HPLC profiles of extracts under different salinities and the α-GG standard; (**B**) Overlay of the ^1^H NMR spectra acquired at 700.185 MHz. The HOD region is shaded. (**C**) Overlay of the ^13^C NMR spectra acquired at 176.080 MHz. Spectra were baseline centered and independently normalized for visual comparison. A single constant referencing correction (−1.298 ppm) was applied to the sample ^13^C axis; no peak-specific alignment or spectral warping was performed. The asterisk indicates a marked signal in the standard spectrum.

HPLC analysis was first performed to examine whether GG was present in the algal extracts obtained under different salinity treatments. The authentic 2-O-α-D-GG standard showed a major peak at 11.08 min, while the algal extracts exhibited a corresponding peak at approximately 11 min. This retention-time match indicated that a GG-like compound was produced in the cells under salt stress (Figure 1A). To further determine its configuration and linkage type, the purified compound was analyzed by ^1^H and ^13^C NMR spectroscopy and directly compared with an authentic 2-O-α-D-GG standard (Figure 1B,C). In the ^1^H NMR spectrum, the product displayed a characteristic anomeric proton signal at δH 5.12 as a doublet with a coupling constant of J = 3.9 Hz. The remaining proton signals were mainly distributed between δH 3.4 and 3.9 ppm, corresponding to the glucosyl ring protons and the glycerol backbone protons. The small anomeric coupling constant was consistent with an α-configured glucopyranoside, whereas a β-glucopyranoside would typically show a larger J1,2 value of approximately 8 Hz. The ^13^C NMR spectrum showed nine major resonances at δC 97.8, 78.7, 72.9, 71.9, 71.5, 69.5, 61.4, 60.4, and 60.3 ppm, consistent with a monoglucosylglycerol skeleton comprising six glucose carbons and three glycerol carbons. The anomeric carbon signal at δC 97.8 supported the presence of an α-glycosidic linkage. In addition, the downfield-shifted glycerol C-2 signal at δC 78.7, compared with free glycerol, indicated that the glucosyl group was attached to the central carbon of glycerol, thereby establishing the 2-O-linkage. Overlaying the product spectra with those of the authentic 2-O-α-D-GG standard showed a peak-for-peak match in both the ^1^H and ^13^C spectra. The ^13^C chemical shifts in the product agreed with those of the standard within ≤0.01 ppm, and the anomeric 1H doublet showed the same coupling constant. No additional resonances attributable to alternative anomers or regioisomers were observed. In particular, no extra signal was detected in the anomeric carbon region around δC 100–108 ppm, where a β-anomeric carbon would be expected. Taken together, these results confirmed that the purified product was 2-O-α-D-GG.

### 2.2. Effects of Culture Conditions on Biomass Accumulation of SCSIO-45682

**Figure 2:**
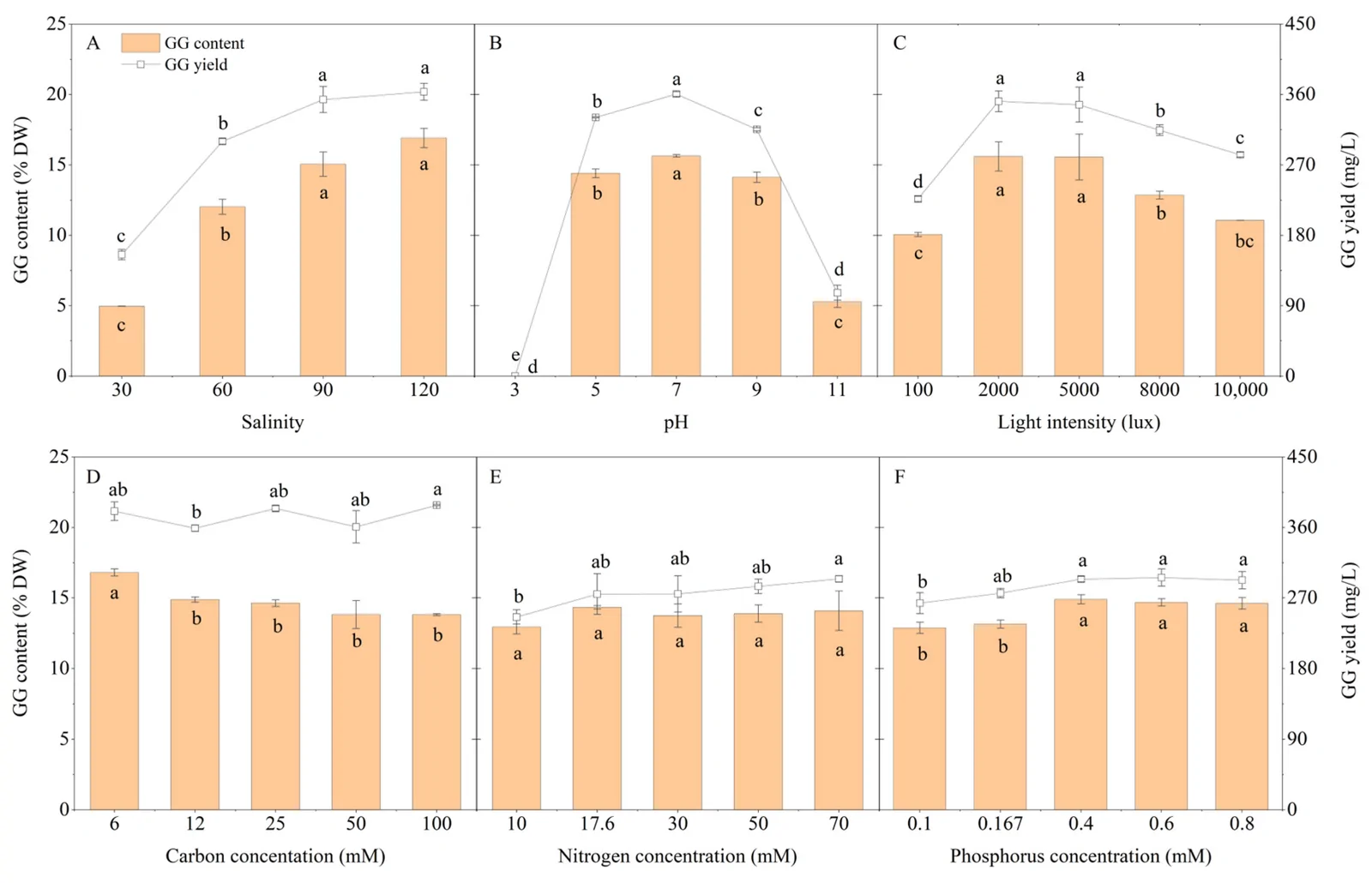
Effects of culture conditions on biomass accumulation in *C*. *aponinum* SCSIO-45682. (**A**) Salinity, (**B**) initial pH, (**C**) light intensity, (**D**) carbon concentration (NaHCO_3_), (**E**) nitrogen concentration (NaNO_3_), and (**F**) phosphorus concentration (NaH_2_PO_4_). The initial inoculum density was OD_750_ = 6.0 ± 0.5, and biomass concentration was determined after 3 days of cultivation. Data represent mean ± SD (*n* = 3). Different lowercase letters above the bars indicate significant differences among treatments within each panel (*p* < 0.05), whereas the same letter indicates no significant difference.

As salinity increased from 30 to 120 ppt, the biomass concentration of SCSIO-45682 showed a gradual decline (Figure 2A). The maximum biomass concentration of 3.13 g/L was achieved at 30 ppt, which then decreased significantly (*p* < 0.05) to 2.35 g/L and 2.15 g/L at 90 and 120 ppt, respectively. SCSIO-45682 exhibited a remarkably wide pH tolerance (pH 3–11). The biomass concentration of SCSIO-45682 showed no significant difference across a pH range of 5 to 9 (*p* > 0.05), with a maximum concentration of approximately 2.28 g/L. At pH 3 and pH 11, the biomass concentration was 2.10 and 2.01 g/L, respectively. These values correspond to significant reductions of 8.75% and 12.57% compared with the maximum biomass concentration of 2.28 g/L (*p* < 0.05). The biomass concentration increased progressively with light intensity, rising from 2.45 g/L at 100 lux to the maximum of 2.78 g/L at 10,000 lux. No photoinhibitory effect was observed within the tested light intensity range (100–10,000 lux).

Among the three nutrients in this study (nitrogen, phosphorus, and carbon), carbon concentration was the most significant factor influencing the growth of SCSIO-45682, with phosphorus and nitrogen exhibiting comparatively minor effects. The biomass concentration increased in an approximately linear manner as carbon concentrations rose from 6 to 100 mM, reaching the maximum of 2.81 g/L. The biomass concentration showed no significant difference under different nitrogen concentrations (*p* > 0.05). The biomass concentration of SCSIO-45682 remained around 2.00 g/L under different phosphorus concentrations. Low phosphorus (0.1–0.167 mM) treatments produced slightly higher biomass than medium and high phosphorus (0.4–0.8 mM) levels (*p* < 0.05).

### 2.3. Effects of Culture Conditions on GG Accumulation of SCSIO-45682

**Figure 3:**
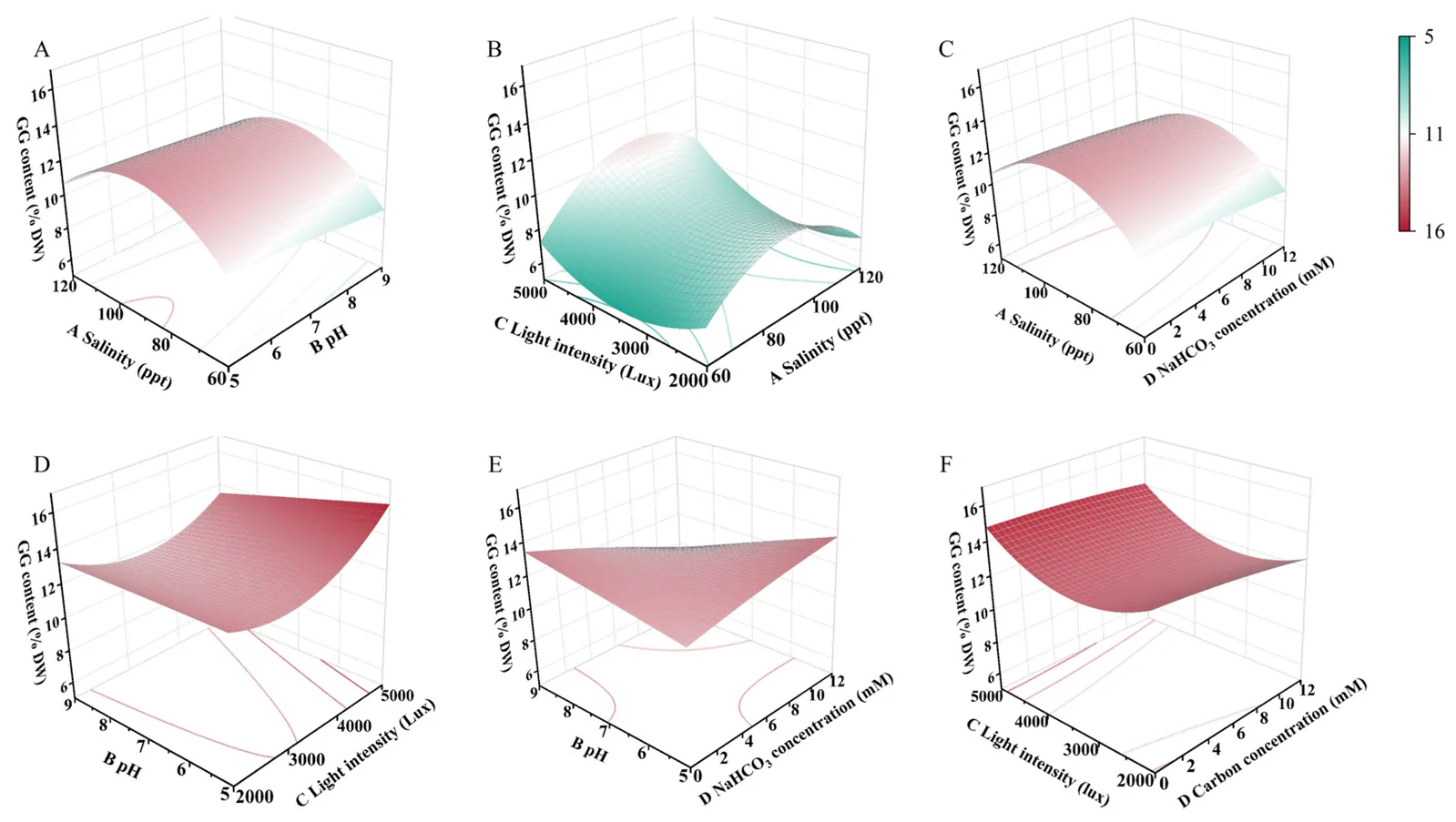
Effects of culture conditions on GG biosynthesis in *C. aponinum* SCSIO-45682. Intracellular GG content (% DW, bars) and volumetric GG yield (mg/L, lines with open square symbols) were determined under varying conditions of (**A**) salinity, (**B**) pH, (**C**) light intensity, (**D**) carbon concentration (NaHCO_3_), (**E**) nitrogen concentration (NaNO_3_), and (**F**) phosphorus concentration (NaH_2_PO_4_). Data represent mean ± SD (n = 3). Different lowercase letters above the bars indicate significant differences among treatments within each panel (*p* < 0.05), whereas the same letter indicates no significant difference.

An increase in salinity from 30 ppt to 120 ppt was accompanied by a 3.41-fold rise in GG content, from 4.95% DW to 16.90% DW (Figure 3A). GG content increased sharply from 30 to 90 ppt (+10.10% DW) but showed no significant further increase from 90 to 120 ppt (*p* > 0.05), indicating that GG accumulation had effectively plateaued by 90 ppt. Within the pH range in the study (pH 3–11), the maximum GG content (15.64% DW) and yield (360.53 mg/L) were obtained at pH 7. GG yield decreased by 70.47% to 106.45 mg/L at pH 11, and GG content was nearly undetectable at pH 3. GG content and yield increased from 100 to 2000 lux, remained at similarly high levels between 2000 and 5000 lux, and then decreased at higher light intensities (8000–10,000 lux). The highest GG content (15.60% DW) and yield (351.19 mg/L) were observed at 2000 lux, although no significant difference was detected between 2000 and 5000 lux (*p* > 0.05). Compared with the maximum at 2000 lux, GG content decreased by approximately 35.55%, 17.63%, and 29.07% at 100, 8000, and 10,000 lux, respectively (*p* < 0.05).

GG accumulation of SCSIO-45682 showed distinct responses to nutrient availability. As carbon concentration increased from 6 to 100 mM, GG content decreased from 16.80% DW to 13.80% DW, representing a reduction of 17.92% (Figure 3C). However, the yield increased to 388.45 mg/L at 100 mM carbon. Nitrogen concentration had no significant effect (*p* > 0.05) on GG content across the tested range (10–70 mM), with the maximum of 14.33% DW observed at 17.6 mM. As phosphorus concentration increased from 0.1 to 0.4 mM, GG content increased significantly by 15.61% (*p* < 0.05), from 12.88% DW to 14.89% DW. No significant difference (*p* > 0.05) in GG content was observed at phosphorus concentrations above 0.4 mM.

### 2.4. Optimization of Culture Conditions for GG Accumulation by RSM

The quadratic polynomial model accurately described the relationship between the culture conditions and GG accumulation based on ANOVA analysis (Table 2). The model showed high statistical significance with *F* = 28.36 and *p* < 0.0001, while the non-significant Lack of Fit (*p* = 0.3895) validated the adequacy of the model. The coefficient of determination (R^2^ = 0.9659) indicated that 96.59% of response variability was explained by the model. The Adjusted R^2^ (0.9319) and Predicted R^2^ (0.8350) showed reasonable agreement (difference = 0.097 < 0.2), indicating acceptable but not exceptional predictive capacity. A difference of 0.097 suggested that while the model reliably described the experimental design space, its predictive accuracy for conditions outside the tested range might be limited, making validation experiments essential. The Adequate Precision value was 20.329, which was far higher than the critical threshold of 4, indicating that the model possessed a favorable signal-to-noise ratio and was reliable for exploring the design space (Table 2).

All four individual factors exerted significant effects on GG content, including salinity (A), pH (B), light intensity (C) and carbon concentration (D), all of which reached significant levels at *p* < 0.05. Beyond individual effects, several interaction terms proved statistically significant: salinity and light intensity (AC), pH and light intensity (BC), and pH and carbon concentration (BD). The BD interaction exhibited particularly strong significance (*F* = 31.45, *p* < 0.0001), indicating a substantial interactive effect between pH and carbon concentration on GG content. Additionally, the quadratic terms for salinity (A^2^) and light intensity (C^2^) were highly significant (*F* = 166.02 and 73.82, respectively), confirming the non-linear nature of these responses and the existence of distinct optima rather than linear relationships.

**Figure 4:**
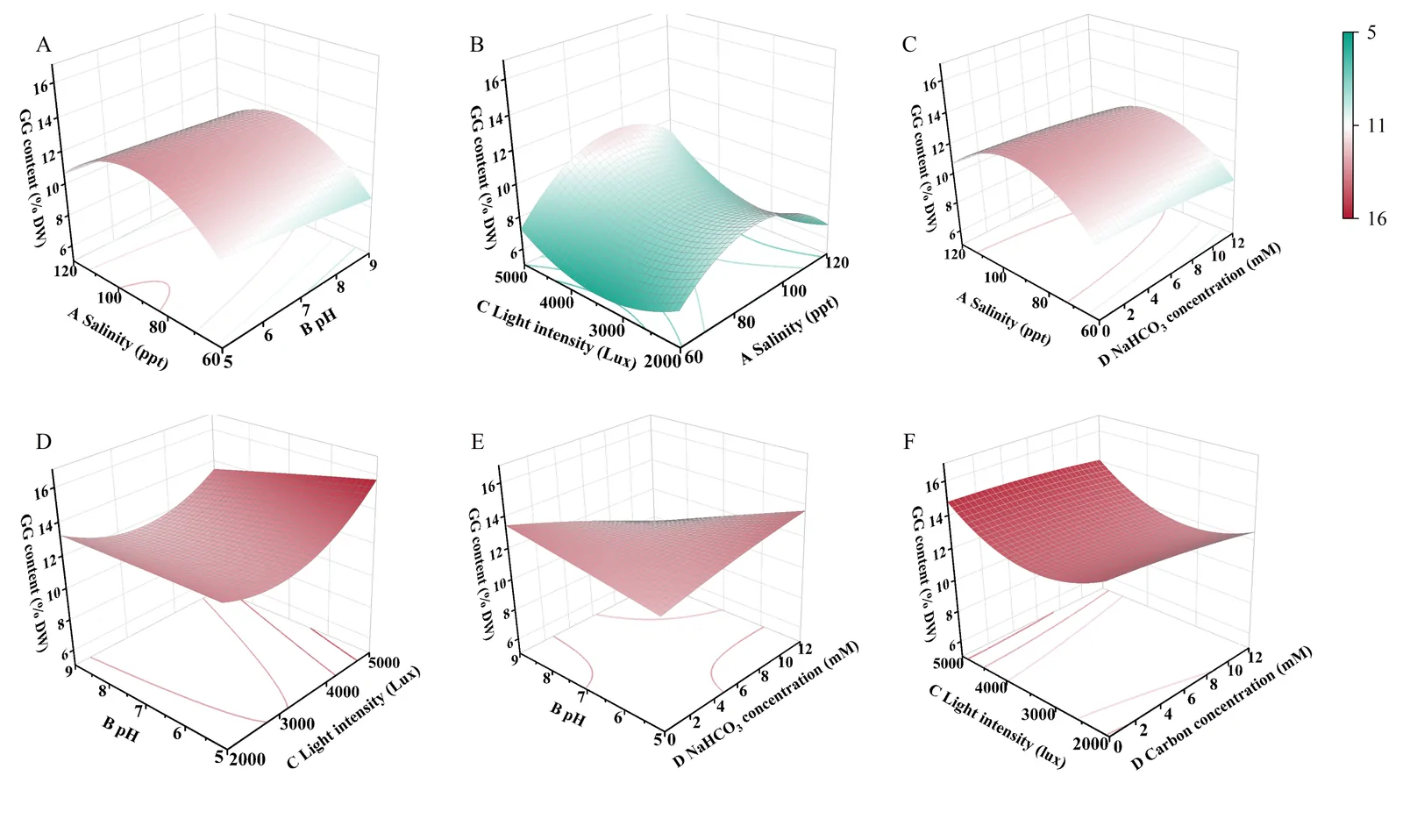
3D response surface and contour plots showing variable interactions on GG content in *C*. *aponinum* SCSIO-45682. (**A**) salinity and pH; (**B**) salinity and light intensity; (**C**) salinity and carbon concentration; (**D**) pH and light intensity; (**E**) pH and carbon concentration; and (**F**) light intensity and carbon concentration. In each graph, the 3D surface represents the predicted GG content, while the 2D contour plot projected on the base visualizes the shape of the interaction. Non-displayed variables were held at their center levels (0 level). The color gradient from green to red indicates the transition from low to high GG content (% DW).

Three-dimensional response surface plots provided a visual representation of the interactions among the tested factors (Figure 4). The salinity × light intensity interaction showed that maximum GG content was achieved at 90 ppt salinity combined with 5000 lux (15.82% DW), whereas GG content declined as salinity shifted toward either 60 ppt or 120 ppt, or as light intensity decreased to 2000 lux (Figure 4B). The pH × light intensity interaction revealed that the optimal light intensity for GG content varied with pH (Figure 4D). At pH 5, the maximum GG content (15.82% DW) was obtained at 5000 lux, whereas the peak GG level at pH 9 (13.61% DW) was achieved at only 2000 lux. This indicated that pH changed the optimal light requirement for GG accumulation. The pH × carbon concentration interaction exhibited an opposite response pattern (Figure 4E). At pH 5, increasing carbon concentration from 0 to 12 mM enhanced GG content from 11.43% to 13.94% DW, whereas at pH 9, the same increase reduced GG content from 13.09% to 10.13% DW.

### 2.5. Verification of RSM Optimization and Biochemical Composition Analysis

**Figure 5:**
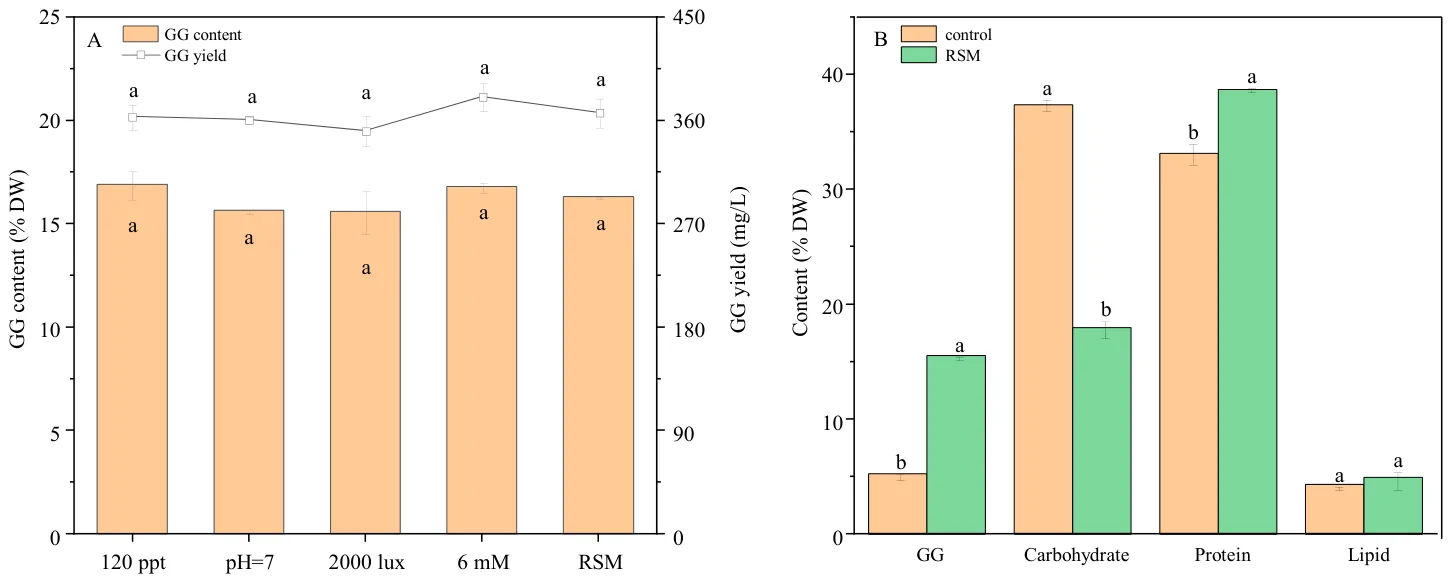
Verification of RSM optimization and comparison of biochemical components. (**A**) Differences in GG content and yield under single-factor maxima and RSM optimal culture conditions. (**B**) Biochemical contents of *C*. *aponinum* SCSIO-45682 cultured under the control and RSM-optimized conditions. Data are presented as mean ± SD (n = 3). Different lowercase letters denote significant differences at *p* < 0.05.

GG accumulation under the RSM-predicted conditions was compared with the maximum values obtained in the single-factor experiments (Figure 5A). In the single-factor experiments, the highest GG contents were 16.90% DW at 120 ppt salinity, 15.64% DW at pH 7, 15.60% DW at 2000 lux, and 16.80% DW at 6 mM NaHCO_3_. Under the RSM-predicted conditions (96 ppt salinity, pH 5.0, 5000 lux, and 7.0 mM NaHCO_3_), GG content reached 16.30% DW, with a volumetric yield of 366.75 mg/L. The RSM-derived GG content was slightly lower than the numerical maximum observed in the single-factor salinity experiment at 120 ppt (16.90% DW), whereas the corresponding volumetric yield was slightly higher than that obtained at 120 ppt (363.31 mg/L).

Biochemical composition under the optimized conditions was further compared with that under the control (30 ppt, pH 7.0, and 12 mM carbon) (Figure 5B). Total carbohydrate content decreased significantly (*p* < 0.05) by 51.93%, from 37.36% DW to 17.96% DW. In contrast, protein content increased significantly (*p* < 0.05) by 16.76%, from 33.12% DW to 38.67% DW. Total lipid content showed no significant difference (*p* > 0.05) between the optimized conditions (4.89% DW) and the control (4.29% DW). Notably, GG content increased 3.29-fold under the optimized conditions based on RSM, rising from 4.95% DW to 16.30% DW.

### 2.6. Radical-Scavenging Activity of the GG-Containing Crude Extract

**Figure 6:**
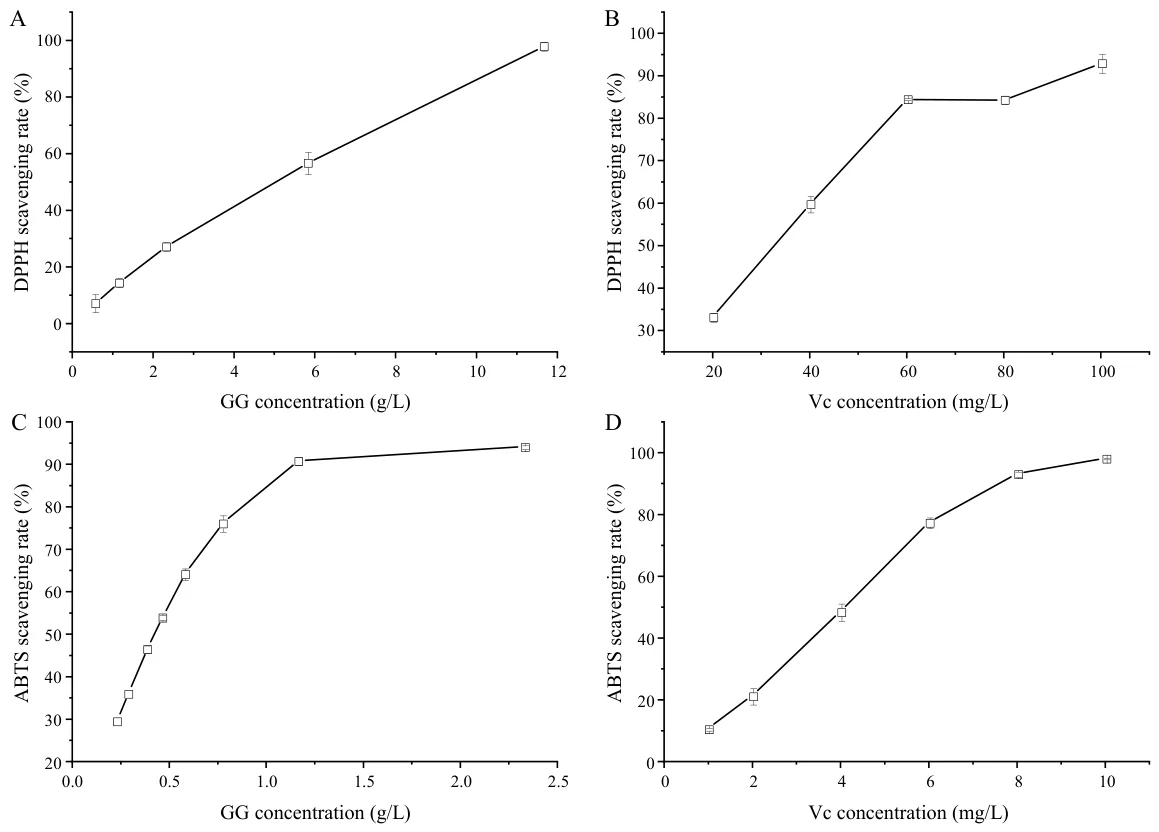
Radical-scavenging activity of the GG-containing crude extract and ascorbic acid (vitamin C, Vc). (**A**) DPPH radical-scavenging activity of the GG-containing crude extract at different GG concentrations; (**B**) DPPH radical-scavenging activity of Vc at different concentrations; (**C**) ABTS radical-scavenging activity of the GG-containing crude extract at different GG concentrations; and (**D**) ABTS radical-scavenging activity of Vc at different concentrations. Data are presented as mean ± SD.

The radical-scavenging activity of the GG-containing crude extract was evaluated using DPPH and ABTS assays, with Vc used as the positive control. In the DPPH assay, the GG-containing crude extract showed concentration-dependent radical-scavenging activity. As the GG concentration in the crude extract increased from 0.58 to 11.66 g/L, the DPPH scavenging rate increased from 7.22% to 97.78% (Figure 6A). The scavenging activity exceeded 50% when the GG concentration was between 2.33 and 5.83 g/L. Based on linear interpolation, the apparent IC_50_ of the crude extract, expressed on the basis of GG concentration, was 5.04 g/L. In comparison, Vc exhibited stronger DPPH radical-scavenging activity, with the scavenging rate increasing from 33.22% at 20 mg/L to 92.84% at 100 mg/L. The IC_50_ value of Vc was 32.65 mg/L. In the ABTS assay, the GG-containing crude extract also exhibited a clear concentration-dependent scavenging effect. As the GG concentration in the crude extract increased from 0.23 to 2.33 g/L, the ABTS scavenging rate increased from 29.77% to 94.18% (Figure 6C). The scavenging rate reached 54.13% at a GG concentration of 0.47 g/L and exceeded 90% at 1.17 g/L. The apparent IC_50_ of the crude extract, expressed on the basis of GG concentration, was 0.42 g/L. Vc showed strong ABTS radical-scavenging activity, with the scavenging rate increasing from 10.83% at 1 mg/L to 98.24% at 10 mg/L. The IC_50_ value of Vc was 4.09 mg/L. These results indicated that the GG-containing crude extract exhibited measurable DPPH and ABTS radical-scavenging activities, with a stronger response observed in the ABTS assay than in the DPPH assay.

## 3. Discussion

The GG product extracted from *C*. *aponinum* SCSIO-45682 was structurally confirmed as 2-O-α-D-GG, indicating that this strain naturally produces the commercially valuable α-GG isomer through photoautotrophic cultivation. The subsequent single-factor experiments and RSM optimization showed that GG accumulation was strongly regulated by culture conditions [3,6]. Salinity and light intensity were identified as the main factors regulating GG accumulation, with salinity acting directly through osmotic pressure and light intensity acting indirectly through photosynthetic metabolism. It is also important to note that GG content and volumetric yield did not always follow the same trend. The efficient GG production in SCSIO-45682 requires a balance between osmotic induction, photosynthetic metabolism, biomass accumulation, and intracellular carbon allocation.

Among all the influencing factors tested in the present study, salinity had the most significant effect on GG accumulation in *C*. *aponinum* SCSIO-45682. GG content increased 3.41-fold from 30 ppt to 120 ppt. High salinity reduced external water potential and promoted osmotic stress, while GG accumulation could maintain intracellular osmotic balance without disrupting cellular metabolism [5,29]. Although inorganic ions can contribute to osmotic adjustment, their excessive accumulation may disturb cellular metabolism. In contrast, GG provides a more compatible and stable strategy for long-term osmotic regulation [5]. Similar salinity-dependent GG accumulation was reported in several cyanobacteria (Table 1). In *Arthrospira maxima*, GG increased from undetectable levels to 15.29% DW as NaCl concentration increased from 0 to 900 mM [17]. Notably, under photoautotrophic cultivation with 600 mM NaCl, *A*. *maxima* achieved a GG content of 15.77% DW and a volumetric yield of 189.99 mg/L (Table 1). In comparison, SCSIO-45682 achieved a similar GG content at 90 ppt (15.05% DW) but a substantially higher volumetric yield of 353.53 mg/L, suggesting that biomass accumulation also contributed to its higher volumetric GG production. In *Synechococcus* sp. PCC 7002, GG was rapidly accumulated under 500 mM NaCl, reaching 2.14% DW after 10 h [16]. In the present study, GG content increased by 10.10% DW from 30 to 90 ppt, with no significant further increase from 90 to 120 ppt (*p* > 0.05, Figure 3A), indicating that GG accumulation had effectively reached the level required for osmotic balance within this salinity range.

Light intensity was the second most influential factor affecting GG synthesis, following salinity. GG content reached its highest value at 2000 lux (15.60% DW). However, when the light intensity was further increased to 8000 and 10,000 lux, GG content decreased by 17.63% and 29.07%, respectively (Figure 3C), whereas biomass accumulation was not adversely affected (Figure 2C). These results indicate that excessive light intensity was unfavorable for GG accumulation. A possible explanation is that excessive irradiance activates photoprotective responses in cyanobacteria. Under high-light conditions, cells may enhance the synthesis of reducing and antioxidant compounds, such as secondary carotenoids and unsaturated fatty acids, to scavenge reactive oxygen species [30]. The biosynthesis of these compounds requires metabolic precursors and reducing power, including GAP, acetyl-CoA, Glu-6-P, and NADPH [31,32]. This creates competition with GG biosynthesis for shared substrates. In addition, ADP-glucose, one of the substrates for GG synthesis, is also a key precursor for the synthesis of storage carbohydrates such as cyanobacterial glycogen [33]. Under excessive light conditions, the cyanobacterial cells prioritize the synthesis of storage compounds for carbon and energy reservation. This would further intensify the competition for ADP-glucose, thereby inhibiting GG synthesis and ultimately reducing intracellular GG content [34]. Similar regulatory effects of light intensity on GG accumulation in cyanobacteria have also been reported in previous studies. Using D_2_O stable isotope labeling, Baran et al. [35] demonstrated rapid turnover of GG and disaccharides during the light phase in cyanobacteria and that carbon from GG can be redistributed into storage carbon pools such as glycogen. This indicates a dynamic interplay between GG and storage carbohydrates. In addition, transcriptomic analysis of *S*. *elongatus* UTEX 2973 showed that high light significantly accelerated cyanobacterial glycogen accumulation, suggesting that fixed carbon is preferentially directed toward storage polysaccharides rather than compatible solutes such as GG [36]. The molecular basis of this carbon competition has been clarified in *Synechocystis* sp. PCC 6803. GG synthase (GgpS) and glycogen synthase (GlgA) share ADP-glucose as a common precursor. When *glgA* was disrupted, ADP-glucose accumulated substantially and was redirected toward GG biosynthesis [33,37]. Taken together, these findings suggest that, under excessive irradiance, carbon flux in cyanobacterial cells is preferentially allocated to glycogen accumulation and the synthesis of photoprotective compounds. This metabolic redistribution may represent an important mechanism underlying the decrease in GG content observed in SCSIO-45682 under high light conditions.

Compared with salinity and light intensity, pH, nitrogen, phosphorus, and carbon concentration had weaker effects on GG accumulation in SCSIO-45682. In the pH range of 5–9, GG content reached the highest value at pH 7 (15.64% DW). However, the overall change was smaller than that under different salinity conditions. This result may be explained for two reasons. First, the change in H^+^ concentration within this pH range was much smaller than the change in ionic strength caused by salinity. Therefore, pH may not act as a strong osmotic stress factor like salinity [38]. Second, cyanobacteria usually have strong pH homeostasis systems. They can maintain stable intracellular pH and carbon fixation through CO_2_-concentrating mechanisms (CCM) and Na^+^/H^+^ antiporter systems [39,40]. This helps maintain the supply of precursors for GG synthesis and the activity of related enzymes. As a result, GG content changed only slightly within this pH range. Within the tested nitrogen and phosphorus ranges, neither factor had a significant effect on GG content. This indicates that nitrogen and phosphorus were not limiting factors for GG synthesis under these conditions. As carbon concentration increased, GG content decreased from 16.80% DW to 13.80% DW. However, GG yield increased to 388.45 mg/L at 100 mM carbon because biomass increased. This result shows a decoupling between GG content and GG yield. Carbon mainly increased GG yield by promoting biomass accumulation. It did not directly enhance the cellular capacity for GG synthesis [17,41]. These results show that nutritional factors and salinity played different roles in GG formation. Nitrogen, phosphorus, and carbon supply were not enough to strongly induce GG accumulation. Their main role was to support cell growth and basic metabolism. Therefore, they affected GG yield mainly through changes in biomass. In contrast, salinity changed the osmotic pressure between the inside and outside of the cells. Thus, salinity was the main signal that induced GG accumulation.

Response surface analysis showed that GG accumulation was not a simple additive effect of individual factors, but rather was synergistically regulated by significant interactive factors (Table 2, Figure 4). Salinity and light intensity showed a synergistic enhancement pattern (Figure 4B). Moderate salt stress at 90 ppt induced GG biosynthesis, while sufficient light intensity at 5000 lux was required to sustain photosynthesis, thereby continuously providing carbon skeletons and reducing power for GG production [42,43]. The pH × light intensity interaction indicated that pH altered the light-intensity requirement for GG accumulation. Under mildly acidic conditions, higher light intensity was more favorable for GG accumulation, whereas under alkaline conditions, the optimal light intensity shifted to a lower level (Figure 4D). The pH × carbon concentration interaction showed an inverse regulatory pattern: carbon supplementation promoted GG accumulation at pH 5 but inhibited GG accumulation at pH 9 (Figure 4E). These observations collectively corroborate the pH sensitivity of cyanobacterial CCM [40]. Under mildly acidic conditions, the increased proportion of CO_2_/H_2_CO_3_ may raise the energetic cost of inorganic carbon enrichment. In this case, external carbon supplementation may help replenish inorganic carbon supply and provide precursors for GG biosynthesis [39]. In contrast, under alkaline conditions at pH 9, inorganic carbon mainly exists as HCO_3_^-^ [44], which is favorable for maintaining the intracellular inorganic carbon pool through the CCM [45]. However, high light intensity exacerbates the reductive pressure on the photosynthetic electron transport chain, forcing cellular metabolism toward photoprotective pathways and thereby suppressing GG synthesis [31,32]. In addition, the extra carbon source may be preferentially directed toward storage polysaccharides or growth-related metabolism rather than the GG biosynthetic pathway [33]. The biochemical composition under the optimized conditions further supported the possibility of metabolic redistribution. Although total carbohydrate content decreased markedly, GG content increased by 3.29-fold. This pattern suggests that the optimized stress combination may have shifted part of the cellular carbohydrate pool toward compatible solute accumulation. These results indicate that GG accumulation is jointly controlled by salt stress, light energy supply, inorganic carbon utilization, and pH homeostasis. This also suggests that future process optimization should be based on the type and strength of factor interactions rather than the simple combination of optimal single-factor levels.

To evaluate the production competitiveness and application potential of GG produced by SCSIO-45682, the RSM-optimized results were compared with previously reported GG production systems (Table 1). Under the RSM-optimized conditions, the GG content of SCSIO-45682 reached 16.30% DW, with a yield of 366.75 mg/L. To our knowledge, this represents the highest GG content reported for wild-type cyanobacteria under photoautotrophic cultivation. This content was higher than that reported for *Arthrospira maxima* under photoautotrophic conditions (15.77% DW), and the GG yield was 1.93- and 4.12-fold higher than those obtained by *A*. *maxima* under photoautotrophic (189.99 mg/L) and mixotrophic (89.00 mg/L) conditions, respectively [17]. The yield also exceeded that of wild-type *Synechocystis* sp. PCC 6803, which is approximately 100 mg/L [18]. These results indicate that through multi-factor synergistic optimization, SCSIO-45682 exhibits a significant advantage in GG accumulation among wild-type cyanobacteria. Although genetically engineered cyanobacteria, engineered heterotrophic bacteria, and enzymatic or whole-cell catalytic systems can achieve much higher GG yield, these systems usually depend on genetic modification, external organic substrates, or additional catalytic processes. By contrast, wild-type SCSIO-45682 provides a non-genetically modified and photoautotrophic strategy for GG production, using inorganic carbon and light as the main inputs. In addition, the GG synthesized through the native cyanobacterial GgpS/GgpP pathway is naturally 2-O-α-D-GG, which may reduce the need for regioisomer separation compared with some enzymatic transglycosylation systems. Beyond production performance, the GG-containing crude extract showed concentration-dependent DPPH and ABTS radical-scavenging activities. However, because the extract was not further purified and its purity was not quantitatively determined, contributions from co-extracted pigments, phenolic compounds, or other antioxidant constituents cannot be excluded. Therefore, further studies using purified 2-O-α-D-GG are required to determine the antioxidant activity attributable specifically to GG. Overall, the advantage of SCSIO-45682 lies not in achieving the highest absolute yield among all GG production systems, but in combining wild-type photoautotrophic production, natural 2-O-α-D-GG formation, and relatively high intracellular GG accumulation. These features support SCSIO-45682 as a promising chassis for the green biomanufacturing of natural GG.

## 4. Materials and Methods

### 4.1. Algal Strain Source and Culture Conditions

The cyanobacterial strain *Cyanobacterium aponinum* SCSIO-45682 used in this study was isolated from the open raceway pond of marine green alga *Picochlorum* sp. located in Tianya Town, Sanya City, Hainan Province, China (109°19′38″ E, 18°18′31″ N). The strain is currently deposited at the China General Microbiological Culture Collection Center (CGMCC, Beijing, China) under the accession number CGMCC No. 19697.

The basal cultivation of the algal strain was conducted in autoclaved f/2 medium [26]. Initial inoculation was performed in column photobioreactors (the South China Sea Institute of Oceanology, Guangzhou, China) maintained at 25 °C with a light intensity of 8000 lux and aeration containing 1% CO_2_. When the algal culture reached late exponential growth phase (OD_750_ = 6–7), cells were harvested for subsequent single-factor and optimization experiments. Unless otherwise specified for experimental variables, all experiments were conducted in 250 mL Erlenmeyer flasks (Changde BKMAM Biotechnology Co., Ltd., Changde, China) with a working volume of 50 mL. The initial inoculum density was controlled at OD_750_ = 6.0 ± 0.5, and cultures were maintained under static conditions at 25 ± 2 °C with a light intensity of 5000 lux and a photoperiod of L:D = 24:0. In subsequent single-factor experiments, only the tested variable was changed, while other conditions remained constant. Flasks were manually shaken three times daily at fixed intervals and randomly repositioned to eliminate edge effects. All experimental treatments were performed in triplicate with a cultivation period of 3 days. The 3-day endpoint was selected based on a preliminary time-course experiment, in which GG content increased rapidly during the first 3 days and approached a plateau thereafter. At the end of each experiment, algal growth status, compatible solute content, and biomacromolecule content were measured.

### 4.2. Single-Factor Optimization Design

To investigate the effects of different conditions on the physiological metabolism of *C*. *aponinum* SCSIO-45682, six single-factor experiments were conducted:

(1) Salinity: pre-cultured algal suspensions at late exponential phase were centrifuged (8000 rpm, 5 min) and the supernatant was discarded. Cells were washed and resuspended in fresh f/2 medium. Different proportions of NaCl were added to the f/2 basal medium to adjust salinity to 30 ppt, 60 ppt, 90 ppt, and 120 ppt, respectively.

(2) pH: Using f/2 medium as the basal medium, the initial pH was adjusted to 3, 5, 7, 9, and 11 using 0.1 M HCl or 0.1 M NaOH solutions.

(3) Light intensity: five light intensity gradients were established by adjusting the number of LED light sources: 100, 2000, 5000, 8000, and 10,000 lux.

(4) To eliminate interference from intracellular nutrient reserves, algal cells were centrifuged prior to inoculation and subjected to starvation culture for 3 days in f/2 medium depleted of C, N, or P, respectively. Using NaHCO_3_ as the carbon source, five concentration gradients were established: 6 mM (0.5×, representing carbon limitation), 12 mM (control), 25 mM, 50 mM, and 100 mM.

(5) Using NaNO_3_ as the nitrogen source, five concentration gradients were established: 10 mM, 17.6 mM (control), 30 mM, 50 mM, and 70 mM.

(6) Using NaH_2_PO_4_ as the phosphorus source, five concentration gradients were established: 0.1 mM, 0.167 mM (control), 0.4 mM, 0.6 mM, and 0.8 mM.

### 4.3. Response Surface Methodology Optimization Design

Based on the single-factor experimental results, four factors showing significant effects on GG accumulation were selected for response surface optimization: salinity (A), pH (B), light intensity (C), and carbon concentration (D). Intracellular GG content (% DW) was used as the response variable and optimization criterion in the Box–Behnken design (BBD), with the objective of identifying cultivation conditions that maximized GG content. The factor ranges used in the BBD were selected to cover the regions associated with relatively high GG accumulation in the single-factor experiments. For salinity, GG content increased markedly from 60 to 120 ppt, while no significant difference was detected between 90 and 120 ppt (*p* > 0.05), indicating similarly high GG accumulation within this range. Accordingly, 60, 90, and 120 ppt were used as the lower, center, and upper salinity levels, respectively. A four-factor, three-level BBD was performed using Design-Expert 13 software. The coded levels of the independent variables are shown in Table 3. Experimental data were fitted using a second-order polynomial equation with the following formula (1):

(1) $$Y\;=\;\beta_{0}+\sum_{i=1}^{k}\beta_{i}x_{i}+\sum_{i=1}^{k}\beta_{ii}x_{i}^{2}+\sum_{i<j}^{k}\beta_{ij}x_{ij}+\varepsilon$$

where Y is the response value (GG content), β_0_ is the intercept term, βᵢ is the linear coefficient, βᵢᵢ is the quadratic coefficient, βᵢⱼ is the interaction coefficient, xᵢ and xⱼ are independent variables, and ε is the error term. Analysis of variance (ANOVA) was employed to evaluate the significance and goodness of fit of the model.

### 4.4. Biomass Concentration Measurement

A specific volume of algal suspension was harvested and filtered through mixed cellulose ester membrane filters (Φ50 mm, 0.45 μm, Hunan BKMAM Holding Co., Ltd., Changde, China) pre-dried to constant weight at 80 °C. The cells were rinsed three times with deionized water and dried at 80 °C to constant weight. After cooling to room temperature in a desiccator, the filters were weighed, and the cell dry weight (DW, g/L) calculated by mass difference.

### 4.5. GG Extraction and HPLC Determination

1 mL of algal suspension was centrifuged to harvest the cells. The pellet was mixed with 1 mL of 80% ethanol and incubated in a 65 °C water bath for 4 h. The extract was filtered through a 0.45 μm membrane filter and quantified using high-performance liquid chromatography (HPLC; Waters Corporation, Milford, MA, USA). Chromatographic separation was performed on an XBridge^®^ BEH Amide column (5 μm, 4.6 × 250 mm, Waters Corporation, Milford, MA, USA) with 80% acetonitrile as the mobile phase at a flow rate of 1 mL/min. The column temperature was maintained at 25 °C. Quantification was based on a standard curve ranging from 0 to 1000 mg/L.

### 4.6. NMR Structural Identification

HPLC analysis showed that GG eluted at approximately 11 min, and the fraction eluting between 10.5 and 12 min was collected. The obtained GG solution was subjected to vacuum distillation using a rotary evaporator (Yarong RE-2000A, Shanghai, China) to remove acetonitrile and obtain an aqueous solution. The aqueous solution was then freeze-dried using a lyophilizer (Scientz, Ningbo, China) to obtain the crude GG extract. An appropriate amount of the crude GG extract was dissolved in D_2_O to prepare a solution at a concentration of 30 mg/mL, which was transferred into an NMR tube (Chongqing Xinweier Glass Co., Ltd., Chongqing, China) for nuclear magnetic resonance spectroscopy analysis.

NMR spectra were recorded at 298 K on a Bruker Avance 700 MHz spectrometer (^1^H, 700.18 MHz; ^13^C, 176.08 MHz, Bruker BioSpin GmbH, Rheinstetten, Germany) equipped with a 5 mm CP TCI cryoprobe. One-dimensional ^1^H NMR spectra were acquired using the zg30 pulse sequence with a 30° excitation pulse, 2 dummy scans, 8–16 scans, 65,536 acquired points, a spectral width of 20.13 ppm, and a relaxation delay of 1 s. Proton-decoupled ^13^C NMR spectra were acquired using the zgpg30 pulse sequence with power-gated 1H decoupling, 8 dummy scans, 6000–8000 scans, 32,768 acquired points, a spectral width of 249.09 ppm, and a relaxation delay of 1 s. Chemical shifts are reported in ppm. The ^1^H chemical shifts were referenced to the residual HDO signal at δH 4.79 ppm, and ^13^C chemical shifts were calibrated by comparison with the authentic 2-O-α-D-GG standard. Coupling constants (J) are reported in Hz.

### 4.7. Biochemical Composition of Cyanobacterial Biomass

At the end of experiments, algal suspensions were harvested, centrifuged, and lyophilized to obtain algal powder (Scientz, Ningbo, China). Total carbohydrate, protein, and total lipid contents were quantitatively analyzed. Total carbohydrate content was determined using a modified phenol-sulfuric acid method [46]. Algal powder was subjected to acid hydrolysis and extraction with 1 M sulfuric acid solution in an 80 °C water bath. After color development, absorbance was measured at 490 nm using glucose as the standard for calibration curve preparation. Protein content was quantified using the Lowry method [47]. Algal powder was extracted with 0.5 M NaOH solution in an 80 °C water bath, and protein concentration was determined using a Lowry protein assay kit (Beijing Solarbio Science & Technology Co., Ltd., Beijing, China). Total lipid content was determined using a modified Soxhlet extraction method [48]. Algal powder was first treated with 10% DMSO-methanol solution (50 °C, 60 min), followed by extraction with diethyl ether/n-hexane (1:1, *v*/*v*) in an ice bath. The organic phases were combined and dried under nitrogen to constant weight, and total lipid content was calculated by the gravimetric method.

### 4.8. DPPH and ABTS Radical Scavenging Assays

Intracellular metabolites were extracted with 80% ethanol as described in Section 4.5. The resulting ethanol extract was concentrated by rotary evaporation without further HPLC purification and used as the GG-containing crude extract for the radical-scavenging assays. The GG concentration in the crude extract was determined by HPLC as described in Section 4.5, and the extract was subsequently diluted to a series of GG concentration gradients. The purity of the crude extract was not quantitatively determined. For the DPPH assay [49], a 100 μmol/L DPPH working solution was prepared in 95% methanol and mixed with an equal volume of the sample solution. The mixture was incubated in the dark at 30 °C for 30 min, and the absorbance was measured at 517 nm using a microplate reader (BioTek Instruments, Inc., Winooski, VT, USA). For the ABTS assay [50], equal volumes of 7.4 mmol/L ABTS solution and 2.6 mmol/L K_2_S_2_O_8_ solution were mixed and kept in the dark at 4 °C for 12–16 h to generate the ABTS radical cation stock solution. Before use, the ABTS stock solution was diluted approximately 26-fold to obtain an absorbance of 0.70 ± 0.05 at 734 nm. The diluted ABTS working solution was then mixed with an equal volume of the sample solution, incubated at 37 °C for 30 min, and the absorbance was measured at 734 nm using a microplate reader. Ascorbic acid (vitamin C, Vc) was used as the positive control in both assays, and the radical scavenging rate was calculated according to Equation (2).

(2) $$\mathrm{DPPH}/\mathrm{ABTS}\;\mathrm{scavenging}\;\mathrm{rate}\;(\%)=\frac{A_{0}-\left(A_{i}-A_{j}\right)}{A_{0}}\times 100\%$$

where A_0_ represents the absorbance of the blank control group (working solution + solvent), A_i_ represents the absorbance of the sample group (working solution + test sample), and A_j_ represents the absorbance of the sample background tube (test sample + solvent), which was used to correct for the intrinsic absorbance of the sample.

### 4.9. Statistical Analysis

For each treatment, three independent culture replicates were established from the same pre-culture. Each replicate flask was cultivated, sampled, and analyzed independently, and the resulting three values were used for statistical analysis. Because all replicates were derived from a common inoculum, they represent independent culture replicates rather than fully independent biological batches; the reported *p* values should therefore be interpreted as reflecting within-pre-culture variability. Results are expressed as mean ± standard deviation (SD). Statistical analyses were conducted using IBM SPSS Statistics 27. One-way analysis of variance (One-way ANOVA) or two-way analysis of variance (Two-way ANOVA) was employed to compare differences among groups, followed by Tukey’s HSD post hoc test for multiple comparisons. Statistical significance was set at *p* < 0.05. Data visualization was performed using Origin 2021 software.

## 5. Conclusions

In this study, the GG product extracted from *C*. *aponinum* SCSIO-45682 was structurally identified as 2-O-α-D-GG. Increased salinity, moderate light intensity of 2000–5000 lux, and near-neutral pH were favorable for GG accumulation, whereas nitrogen, phosphorus, and carbon concentrations had comparatively weaker effects on intracellular GG content. Response surface methodology further revealed significant interactions among the major cultivation factors and identified conditions that maximized intracellular GG accumulation (96 ppt salinity, pH 5.0, 5000 lux, and 7.0 mM NaHCO_3_). Under these conditions, GG content and volumetric yield reached 16.30% DW and 366.75 mg/L, respectively. The GG-containing crude extract exhibited measurable DPPH and ABTS radical-scavenging activities; however, further studies using purified GG are required to determine whether these activities can be attributed specifically to GG. Overall, these findings establish *C*. *aponinum* SCSIO-45682 as a promising wild-type photoautotrophic platform for the sustainable production and functional application of natural 2-O-α-D-GG. Future studies should focus on clarifying the metabolic regulation and carbon-partitioning mechanisms underlying GG accumulation, further improving volumetric productivity through cultivation and process optimization, and evaluating GG production under larger-scale and outdoor cultivation conditions. In addition, more comprehensive functional and application-oriented assessments will be needed to further evaluate the potential of naturally produced GG in cosmetic, food, and pharmaceutical applications.

## Figures and Tables

**Table 1:** Comparison of GG production across different biological and enzymatic systems.

Strain/System	Type	Strategy	Salinity/Substrate	GG Content (% DW)	Volumetric Yield	Regio-Selectivity	Reference
*C. aponinum* SCSIO-45682	Cyanobacterium (WT)	BBD-RSM Optimization	96 ppt NaCl, pH 5.0, 5000 lux, 7 mM NaHCO_3_	16.30	366.75 mg/L	100% (Natural)	This Study
*Arthrospira maxima*	Cyanobacterium (WT)	Photoautotrophic, NaCl induction	600 mM NaCl	15.77	189.99 mg/L	100% (natural)	[17]
		Mixotrophic, NaCl induction	600 mM NaCl	6.52	89.00 mg/L	100% (natural)	
*Synechocystis* sp. PCC 6803	Cyanobacterium (WT)	Salt stress, batch	600 mM NaCl	NR	~100 mg/L	100% (natural)	[18]
	Cyanobacterium (Engineered)	Gene knockout + gel encapsulation, semicontinuous	600 mM NaCl	NR	1640 mg/L (cumulative)	100% (natural)	
*C*. *glutamicum* ΔotsA IM-glgA (pEKEx3-ggpSP)	Heterotrophic bacterium (Engineered)	Heterologous ggpSP, N-limitation	750 mM NaCl, sucrose	N/A (secreted)	~2000 mg/L	100% (GgpS)	[19]
*E*. *coli* BL21(DE3) (LmSucP)	Enzymatic (whole-cell catalyst)	Freeze–thaw permeabilized cells, SucP catalysis	300 mM sucrose + 1.8 M glycerol	N/A	65 g/L	≥90% (LmSucP)	[21]
*E*. *coli* (LmSPase engineered)	Enzymatic (whole-cell catalyst)	SPase engineering + RBS fine-tuning, fed-batch	Sucrose + glycerol	N/A	351.8 g/L	NR	[22]
*Lb*. *paracasei* (LrSP)	Enzymatic (whole-cell catalyst)	SPase with improved glycerol affinity	1 M sucrose + 1 M glycerol	N/A	203.21 g/L	NR	[23]
In vitro enzymatic (SPase + GGP cascade)	Enzymatic catalysis	Phosphorolysis + transglycosylation	2 M sucrose + 2.4 M glycerol	N/A	452 g/L	100% (GGP)	[20]

NR = not reported; N/A = not applicable. Regioselectivity refers to the proportion of 2-O-α-D-glucosylglycerol (2-αGG) in total GG product.

**Table 2:** Analysis of variance results for the quadratic polynomial model.

Source	Sum of Squares	df	Mean Square	F-Value	*p*-Value	
Model	94.3900	14	6.7400	28.3600	<0.0001	***
A-Salinity	2.3000	1	2.300	9.6900	0.0076	**
B-pH	4.2200	1	4.2200	17.7600	0.0009	***
C-Light Intensity	4.8700	1	4.8700	20.4900	0.0005	***
D-Carbon concentration	3.2000	1	3.2000	13.4500	0.0025	**
AB	0.1892	1	0.1892	0.7957	0.3874	
AC	1.3100	1	1.3100	5.5100	0.0341	*
AD	1.3100 × 10^−7^	1	1.3100 × 10^−7^	4.7580 × 10^−7^	0.9995	/
BC	1.1600	1	1.1600	4.8700	0.0446	*
BD	7.4800	1	7.4800	31.4500	<0.0001	***
CD	0.1150	1	0.1150	0.4839	0.4980	/
A2	39.4700	1	39.4700	166.02000	<0.0001	***
B2	0.0041	1	0.0041	0.0171	0.8980	/
C2	17.5500	1	17.5500	73.8200	<0.0001	***
D2	0.1369	1	0.1369	0.5757	0.4606	/
Residual	3.3300	14	0.2377	/	/	/
Lack of Fit	2.6000	10	0.2602	1.4300	0.3895	/
Pure Error	0.7265	4	0.1816	/	/	/
Cor Total	97.7200	28	/	/	/	/
R^2^	0.9659	/	/	/	/	/
Adjusted R^2^	0.9319	/		/	/	/

Note: * *p* < 0.05, ** *p* < 0.01, and *** *p* < 0.001.

**Table 3:** Variables and coded levels used in the Box–Behnken design.

Variable	Code	Coded Variable Level		
		−1	0	+1
Salinity (ppt)	A	60	90	120
pH	B	5	7	9
Light intensity (lux)	C	2000	3500	5000
NaHCO_3_ concentration (mM)	D	0	6	12

## Data Availability

The data presented in this study are available on request from the corresponding author due to legal reasons.

## Abbreviations

The following abbreviations are used in this manuscript:

GG	Glucosylglycerol
2-O-α-D-GG	2-O-α-D-glucosylglycerol
DW	Dry weight
HPLC	High-performance liquid chromatography
NMR	Nuclear magnetic resonance
RSM	Response surface methodology
BBD	Box–Behnken design
DPPH	2,2-diphenyl-1-picrylhydrazyl
ABTS	2,2′-azino-bis(3-ethylbenzothiazoline-6-sulfonic acid)
Vc	Vitamin C/Ascorbic acid
IC_50_	Half-maximal inhibitory concentration
OD_750_	Optical density at 750 nm
CCM	CO_2_-concentrating mechanism
HDO/HOD	Residual semi-heavy water signal
ADP-glucose	Adenosine diphosphate glucose
GAP	Glyceraldehyde 3-phosphate
Glu-6-P	Glucose 6-phosphate
NADPH	Reduced nicotinamide adenine dinucleotide phosphate

## References

[1] Gerwick W.H., Moore B.S. (2012). Lessons from the Past and Charting the Future of Marine Natural Products Drug Discovery and Chemical Biology. Chem. Biol..

[2] Demay J., Bernard C., Reinhardt A., Marie B. (2019). Natural Products from Cyanobacteria: Focus on Beneficial Activities. Mar. Drugs.

[3] Klähn S., Hagemann M. (2011). Compatible solute biosynthesis in cyanobacteria. Environ. Microbiol..

[4] Pade N., Hagemann M. (2015). Salt Acclimation of Cyanobacteria and Their Application in Biotechnology. Life.

[5] Hagemann M. (2011). Molecular biology of cyanobacterial salt acclimation. FEMS Microbiol. Rev..

[6] Kirsch F., Klähn S., Hagemann M. (2019). Salt-Regulated Accumulation of the Compatible Solutes Sucrose and Glucosylglycerol in Cyanobacteria and Its Biotechnological Potential. Front. Microbiol..

[7] Tan X., Luo Q., Lu X. (2016). Biosynthesis, biotechnological production, and applications of glucosylglycerols. Appl. Microbiol. Biotechnol..

[8] Luo Q., Duan Y., Lu X. (2022). Biological sources, metabolism, and production of glucosylglycerols, a group of natural glucosides of biotechnological interest. Biotechnol. Adv..

[9] Schrader A., Siefken W., Kueper T., Breitenbach U., Gatermann C., Sperling G., Biernoth T., Scherner C., Stäb F., Wenck H. (2012). Effects of Glyceryl Glucoside on AQP3 Expression, Barrier Function and Hydration of Human Skin. Ski. Pharmacol. Physiol..

[10] Sawangwan T., Goedl C., Nidetzky B. (2010). Glucosylglycerol and glucosylglycerate as enzyme stabilizers. Biotechnol. J..

[11] Harada N., Zhao J., Kurihara H., Nakagata N., Okajima K. (2010). Effects of Topical Application of α-d-Glucosylglycerol on Dermal Levels of Insulin-Like Growth Factor-I in Mice and on Facial Skin Elasticity in Humans. Biosci. Biotechnol. Biochem..

[12] Oresajo C., Pillai S., Manco M., Yatskayer M., McDaniel D. (2012). Antioxidants and the skin: Understanding formulation and efficacy. Dermatol. Ther..

[13] Borowitzka L.J., Demmerle S., MacKay M.A., Norton R.S. (1980). Carbon-13 Nuclear Magnetic Resonance Study of Osmoregulation in a Blue-Green Alga. Science.

[14] Reed R.H., Richardson D.L., Warr S.R.C., Stewart W.D.P. (1984). Carbohydrate Accumulation and Osmotic Stress in Cyanobacteria. Microbiology.

[15] Marin K., Huckauf J., Fulda S., Hagemann M. (2002). Salt-Dependent Expression of Glucosylglycerol-Phosphate Synthase, Involved in Osmolyte Synthesis in the Cyanobacterium *Synechocystis* sp. Strain PCC 6803. J. Bacteriol..

[16] Aikawa S., Nishida A., Hasunuma T., Chang J.-S., Kondo A. (2019). Short-Term Temporal Metabolic Behavior in Halophilic Cyanobacterium *Synechococcus* sp. Strain PCC 7002 after Salt Shock. Metabolites.

[17] Huang J., Yao T., Yi S., Sun D., Zhang P., Wang H., Xue X., Zhang R. (2025). Effects of salt concentration and trophic mode on glucosylglycerol and phycocyanin production by *Arthrospira maxima*. Algal Res..

[18] Tan X., Du W., Lu X. (2015). Photosynthetic and extracellular production of glucosylglycerol by genetically engineered and gel-encapsulated cyanobacteria. Appl. Microbiol. Biotechnol..

[19] Roenneke B., Rosenfeldt N., Derya S.M., Novak J.F., Marin K., Krämer R., Seibold G.M. (2018). Production of the compatible solute α-d-glucosylglycerol by metabolically engineered *Corynebacterium glutamicum*. Microb. Cell Fact..

[20] Zhang T., Yang J., Tian C., Ren C., Chen P., Men Y., Sun Y. (2020). High-Yield Biosynthesis of Glucosylglycerol through Coupling Phosphorolysis and Transglycosylation Reactions. J. Agric. Food Chem..

[21] Schwaiger K.N., Cserjan-Puschmann M., Striedner G., Nidetzky B. (2021). Whole cell-based catalyst for enzymatic production of the osmolyte 2-O-α-glucosylglycerol. Microb. Cell Fact..

[22] Duan P., Long M., Zhang X., Liu Z., You J., Pan X., Fu W., Xu M., Yang T., Shao M. (2023). Efficient 2-O-α-D-glucopyranosyl-sn-glycerol production by single whole-cell biotransformation through combined engineering and expression regulation with novel sucrose phosphorylase from Leuconostoc mesenteroides ATCC 8293. Bioresour. Technol..

[23] Cui Y., Xu Z., Yue Y., Kong W., Kong J., Guo T. (2024). 2-O-α-D-glucosyl glycerol production by whole-cell biocatalyst of lactobacilli encapsulating sucrose phosphorylase with improved glycerol affinity and conversion rate. Microb. Cell Fact..

[24] Meng F., Cui H., Wang Y., Li X. (2018). Responses of a new isolated *Cyanobacterium aponinum* strain to temperature, pH, CO_2_ and light quality. J. Appl. Phycol..

[25] Winckelmann D., Bleeke F., Bergmann P., Klöck G. (2015). Growth of *Cyanobacterium aponinum* influenced by increasing salt concentrations and temperature. 3 Biotech.

[26] Chen Z., Li T., Yang B., Jin X., Wu H., Wu J., Lu Y., Xiang W. (2021). Isolation of a novel strain of *Cyanobacterium* sp. with good adaptation to extreme alkalinity and high polysaccharide yield. J. Oceanol. Limnol..

[27] Chen Z., Wu J., Wang N., Li T., Wu H., Wu H., Xiang W. (2024). Isolation, Characterization, Moisturization and Anti-HepG2 Cell Activities of a Novel Polysaccharide from *Cyanobacterium aponinum*. Molecules.

[28] Lin J., Ng I.S. (2021). Production, isolation and characterization of C-phycocyanin from a new halo-tolerant *Cyanobacterium aponinum* using seawater. Bioresour. Technol..

[29] Kempf B., Bremer E. (1998). Uptake and synthesis of compatible solutes as microbial stress responses to high-osmolality environments. Arch. Microbiol..

[30] Muramatsu M., Hihara Y. (2012). Acclimation to high-light conditions in cyanobacteria: From gene expression to physiological responses. J. Plant Res..

[31] Latifi A., Ruiz M., Zhang C.-C. (2009). Oxidative stress in cyanobacteria. FEMS Microbiol. Rev..

[32] Kirilovsky D., Kerfeld C.A. (2016). Cyanobacterial photoprotection by the orange carotenoid protein. Nat. Plants.

[33] Xu Y., Tiago Guerra L., Li Z., Ludwig M., Charles Dismukes G., Bryant D.A. (2013). Altered carbohydrate metabolism in glycogen synthase mutants of *Synechococcus* sp. strain PCC 7002: Cell factories for soluble sugars. Metab. Eng..

[34] Pade N., Mikkat S., Hagemann M. (2017). Ethanol, glycogen and glucosylglycerol represent competing carbon pools in ethanol-producing cells of *Synechocystis* sp. PCC 6803 under high-salt conditions. Microbiology.

[35] Baran R., Lau R., Bowen B.P., Diamond S., Jose N., Garcia-Pichel F., Northen T.R. (2017). Extensive Turnover of Compatible Solutes in Cyanobacteria Revealed by Deuterium Oxide (D_2_O) Stable Isotope Probing. ACS Chem. Biol..

[36] Tan X., Hou S., Song K., Georg J., Klähn S., Lu X., Hess W.R. (2018). The primary transcriptome of the fast-growing cyanobacterium *Synechococcus elongatus* UTEX 2973. Biotechnol. Biofuels.

[37] Díaz-Troya S., López-Maury L., Sánchez-Riego A.M., Roldán M., Florencio F.J. (2014). Redox Regulation of Glycogen Biosynthesis in the Cyanobacterium *Synechocystis* sp. PCC 6803: Analysis of the AGP and Glycogen Synthases. Mol. Plant.

[38] Oren A., Rosenberg E., DeLong E.F., Lory S., Stackebrandt E., Thompson F. (2013). Life at High Salt Concentrations. The Prokaryotes: Prokaryotic Communities and Ecophysiology.

[39] Price G.D., Badger M.R., Woodger F.J., Long B.M. (2008). Advances in understanding the cyanobacterial CO_2_-concentrating-mechanism (CCM): Functional components, Ci transporters, diversity, genetic regulation and prospects for engineering into plants. J. Exp. Bot..

[40] Mangan N.M., Flamholz A., Hood R.D., Milo R., Savage D.F. (2016). pH determines the energetic efficiency of the cyanobacterial CO_2_ concentrating mechanism. Proc. Natl. Acad. Sci. USA.

[41] Aikawa S., Nishida A., Ho S.-H., Chang J.-S., Hasunuma T., Kondo A. (2014). Glycogen production for biofuels by the euryhaline cyanobacteria *Synechococcus* sp. strain PCC 7002 from an oceanic environment. Biotechnol. Biofuels.

[42] Hagemann M., Erdmann N. (1994). Activation and pathway of glucosylglycerol synthesis in the cyanobacterium *Synechocystis* sp. PCC 6803. Microbiology.

[43] Lucius S., Hagemann M. (2024). The primary carbon metabolism in cyanobacteria and its regulation. Front. Plant Sci..

[44] Stumm W., Morgan J.J. (1996). Aquatic Chemistry: Chemical Equilibria and Rates in Natural Waters.

[45] Fang S., Huang X., Zhang X., Zhang M., Hao Y., Guo H., Liu L.-N., Yu F., Zhang P. (2021). Molecular mechanism underlying transport and allosteric inhibition of bicarbonate transporter SbtA. Proc. Natl. Acad. Sci. USA.

[46] Masuko T., Minami A., Iwasaki N., Majima T., Nishimura S.-I., Lee Y.C. (2005). Carbohydrate analysis by a phenol–sulfuric acid method in microplate format. Anal. Biochem..

[47] Prakash Jain B., Pandey S., Goswami S.K., Prakash Jain B., Pandey S., Goswami S.K. (2025). Chapter 39—Protein Estimation by Folin-Ciocalteau (Lowry) Method. Protocols in Biochemistry and Clinical Biochemistry.

[48] Khozin-Goldberg I., Shrestha P., Cohen Z. (2005). Mobilization of arachidonyl moieties from triacylglycerols into chloroplastic lipids following recovery from nitrogen starvation of the microalga *Parietochloris incisa*. Biochim. Biophys. Acta (BBA) Mol. Cell Biol. Lipids.

[49] Wang N., Pei H., Xiang W., Li T., Lin S., Wu J., Chen Z., Wu H., Li C., Wu H. (2023). Rapid Screening of Microalgae as Potential Sources of Natural Antioxidants. Foods.

[50] Venkatesan M., Arumugam V., Pugalendi R., Ramachandran K., Sengodan K., Vijayan S.R., Sundaresan U., Ramachandran S., Pugazhendhi A. (2019). Antioxidant, anticoagulant and mosquitocidal properties of water soluble polysaccharides (WSPs) from Indian seaweeds. Process Biochem..

